# A System of Surgery

**Published:** 1912-03

**Authors:** 


**A System of Surgery.** Edited by C. C. Choyce, B.Sc., M.D. Vol. I. Pp. xxii, 957. London: Cassell & Company, Ltd. 1912.

On looking through the list of contributors to this book one is led to expect a work of considerable merit. A perusal of the first volume in no way disappoints this expectation. It deals chiefly with pathology and general surgery.

In the first hundred pages the reader will find an extremely lucid account of the more important modern discoveries in connection with bacteriology. Those micro-organisms of chief surgical importance are carefully described with their staining and cultural peculiarities, and they are well illustrated by numerous photomicrographs. Such processes as Widal's and Wassermann's reactions are carefully described, and all the necessary technical details are carefully set forth.

Full accounts will be found of local and spinal analgesia. The writer of the latter is evidently quite an enthusiast. In our opinion he does not lay sufficient stress upon the disadvantages of this method as compared with general anæsthesia. He makes no mention of those tragic deaths which mar the record of even the most experienced administrators. Though we must admit similar occurrences when general anæsthetics are given, the proportion is undoubtedly less. Instances are, however, quoted of respiratory failure following Jonnesco's method of inducing high analgesia. In three out of nine cases in which it was tried at the Dreadnought Hospital, Greenwich, alarming symptoms supervened.

After reading Colonel Lambkin's article on syphilis, and noting his strong advocacy of intramuscular injection of mercurial salts, one cannot help wondering that one so seldom sees this method of treatment adopted in the clinic of a general hospital. Is it that both surgeon and patient are "needle shy," or is it that "Rep. mist." is so easy to stamp upon a card? Hydrotherapy as an adjuvant to the usual routine treatment is strongly insisted on. We think this is too often neglected.

Salvarsan has a chapter to itself. The treatment is fully discussed, and the different methods of giving the drug are described in detail. Interesting photographs of patients before and after treatment are appended.

The chapters on military surgery and on radiography are particularly interesting.

Some of the radiograms in the book are beautiful specimens. The illustrations throughout are of a high order of merit. Printing and paper are good, and the book is well spaced and headed. There are few printer's errors. We trust that "injection" and "infection" (p. 681) are not often synonymous.

